# Chemical structure imaging of a single molecule by atomic force microscopy at room temperature

**DOI:** 10.1038/ncomms8766

**Published:** 2015-07-16

**Authors:** Kota Iwata, Shiro Yamazaki, Pingo Mutombo, Prokop Hapala, Martin Ondráček, Pavel Jelínek, Yoshiaki Sugimoto

**Affiliations:** 1 Graduate School of Engineering, Osaka University, 2-1, Yamada-Oka, Suita, Osaka 565-0871, Japan.; 2 The Institute of Scientific and Industrial Research, Osaka University, 8-1 Mihogaoka, Ibaraki, Osaka 567-0047, Japan.; 3 Institute of Physics, Academy of Sciences of the Czech Republic, Cukrovarnická 10/112, Prague 16200, Czech Republic.; 4 Department of Advanced Materials Science, University of Tokyo 5-1-5, Kashiwanoha, Kashiwa, Chiba 277-8561, Japan.

## Abstract

Atomic force microscopy is capable of resolving the chemical structure of a single molecule on a surface. In previous research, such high resolution has only been obtained at low temperatures. Here we demonstrate that the chemical structure of a single molecule can be clearly revealed even at room temperature. 3,4,9,10-perylene tetracarboxylic dianhydride, which is strongly adsorbed onto a corner-hole site of a Si(111)–(7 × 7) surface in a bridge-like configuration is used for demonstration. Force spectroscopy combined with first-principle calculations clarifies that chemical structures can be resolved independent of tip reactivity. We show that the submolecular contrast over a central part of the molecule is achieved in the repulsive regime due to differences in the attractive van der Waals interaction and the Pauli repulsive interaction between different sites of the molecule.

## Abstract (web summary)

Atomic force microscopy is capable of resolving the chemical structure of a single molecule on a surface, usually at low temperatures. Here, the authors demonstrate that the chemical structure of a single molecule strongly adsorbed onto a silicon surface can be determined at room temperature.

## Graphical abstract

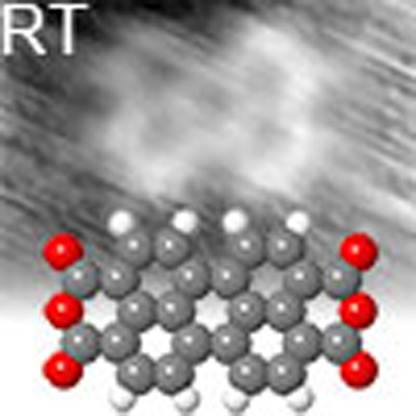

Atomic force microscopy (AFM) with a frequency modulation technique^1^ has proven successful for imaging various sample surfaces at high resolution^2^,^3^. Since the first-reported use of AFM to image the chemical structures of single organic molecules^4^, numerous molecular studies have employed AFM, such as for the structural determination of natural compounds^5^, the imaging of intracharge distributions^6^,^7^, bond order discrimination^8^, discrimination of products after chemical reactions^9^ and intermolecular contrasts^10^,^11^,^12^,^13^. Chemical structures can also be obtained by scanning tunnelling microscopy (STM) using single atoms or molecules below/on the tip as transducers^14^,^15^. Thus far, such studies have been performed at 5 K, except for a few reports at 77 K (refs 11, 16, 17). Most investigators have used the so-called qPlus sensor^18^, in which one free-standing prong of a quartz tuning fork acts as a stiff cantilever, while the other prong is fixed. The short-range interaction force is selectively detected by small cantilever oscillation amplitude (*A*). In addition, functionalized tips, such as the CO-terminated tip, are effective in enhancing spatial resolution^19^.

In this study, we demonstrate that the chemical structure of single molecules can be revealed by AFM even at room temperature (RT). Such RT imaging is challenging because CO tip functionalization is not feasible, and it is relatively hard to maintain a stable-tip state. RT imaging can be achieved by AFM, however, by using optical interferometry with high force sensitivity and soft silicon cantilevers. Submolecular AFM imaging at RT offers great promise for further application.

## Results

### High-resolution AFM imaging of a single molecule

The present experiments were performed by using a custom-built AFM/STM operated at RT under an ultrahigh vacuum^20^. Commercial conductive Si cantilevers were used after Ar ion sputtering treatment. The cantilever was oscillated at relatively large amplitude (*A*∼10 nm) for stable operation. A sharp Si tip is essential to maintain stable-tip states at RT, and to suppress long-range interaction force contributions^21^. The cantilever deflection was detected by an optical interferometer with high sensitivity^22^. The frequency shift (Δ*f*) of the cantilever oscillation was recorded as an AFM signal. The time-averaged tunnelling current was measured from the cantilever while bias voltage (*V*_*S*_) was applied to a sample. *V*_*S*_ was set to compensate contact potential difference (*V*_c.p.d._) for AFM. We investigated PTCDA (3,4,9,10-perylene tetracarboxylic dianhydride) molecules evaporated on a reactive semiconductor surface, that is, the Si(111)-(7 × 7) surface.

**Figure 1:**
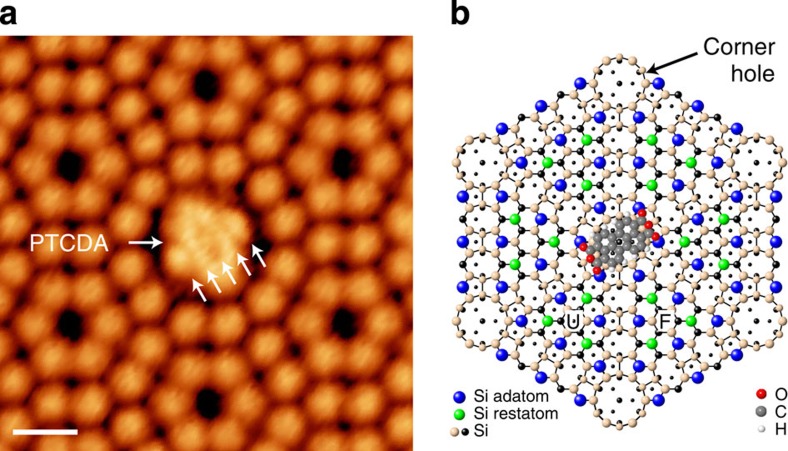
A single PTCDA molecule adsorbed on the Si(111)-(7 × 7) surface. (**a**) An STM image at *V*_*s*_=+500 mV. Scale bar, 1 nm. The five arrows indicate stripes corresponding to a molecular orbital. (**b**) Schematic view of the arrangement of a PTCDA molecule adsorbed onto the surface over a corner-hole site.

We first used STM to investigate the very initial adsorption of PTCDA molecules onto the Si(111)-(7 × 7) surface. Some PTCDA molecules were adsorbed onto corner-hole sites in a well-defined manner, as first reported by Nicoara *et al*.^23^. A typical STM topographic image is shown in Fig. 1a. Characteristic features appear on the molecule in the centre of the image beside the atomic resolution of the Si(111)-(7 × 7) surface. Figure 1b shows the proposed adsorption geometry corresponding to the topographic image in Fig. 1a. The aromatic rings at the centre of the molecule are well detached from the Si substrate, since atoms are missing up to the third layer at the corner-hole sites. In contrast, the carboxyl groups of the molecule strongly bind with dangling bonds of the four corner Si adatoms by partial ionic covalent bonding^23^. The bonds are strong enough to suppress thermal diffusion of the molecule even at RT. In our empty-state STM image, five parallel stripes are imaged in the molecule as indicated by the arrows in Fig. 1a. They were identified as originating from a molecular orbital that was located near the Fermi level and was energetically shifted from the original LUMO+1 and LUMO+2 because of molecule–substrate coupling^23^.

**Figure 2:**
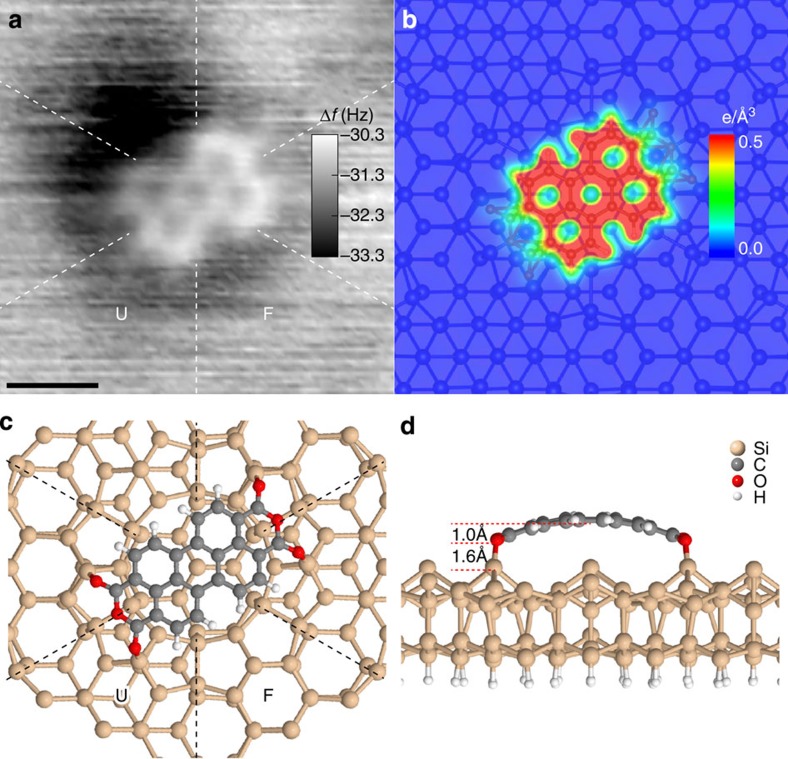
Chemical structure imaging of single molecule by AFM at room temperature. (**a**) Constant-height AFM image of a single PTCDA molecule adsorbed on the Si(111)-(7 × 7) surface. The acquisition parameters were *f*_0_=158,586.6 Hz, *k*=31.9 N/m, *A*=138 Å, *V*_*s*_=*V*_c.p.d._=130 mV, and *Q*=22,000. The scan speed was 3 nm s^-1^. Scale bar, 0.5 nm. (**b**) Calculated map of electron density cut along a plane parallel to the surface. (**c**,**d**) Top and side views of the optimized configuration of the PTCDA molecule adsorbed at a corner-hole site of the Si(111)-(7 × 7) surface.

When a PTCDA molecule was found at a corner-hole site by STM, we switched the imaging mode to a constant-height AFM scan^21^. The tip–surface distances were gradually decreased until the repulsive force became significant on the molecule. In some cases, the contrast was unclear or the tip state changed or the molecule moved away from the original location by the tip before the tip reached the distance for submolecular resolution as shown in Supplementary Fig. 1. Then, the tip was modified by intentionally crashing it into the clean region of the Si surface to obtain stable tips. We then tried again to image the molecule. By repeating these procedures, we successfully obtained AFM images of single molecules, revealing their chemical structure even at RT, as shown in Fig. 2a. Five centre-carbon rings were clearly resolved by the local positive shift of Δ*f* due to the Pauli repulsive force. Thus, AFM and STM clearly offer complementary information, that is, chemical structure and molecular orbital data, respectively. The detailed procedures for high-resolution AFM imaging are described in Methods and Supplementary Fig. 1.

To gain insight into the geometry of the molecule and imaging mechanism, we carried out density functional theory (DFT) calculations. The top and side views of the optimized configuration of the molecule adsorbed on the Si(111)-(7 × 7) surface are shown in Fig. 2c,d, respectively. Oxygen atoms were located above Si adatoms by 1.6 Å. Furthermore, the molecule was bent upward by 1.0 Å. This may have been due to the directionality of the Si-dangling bonds that are bound with the oxygen atoms. The central part of the molecule thus protruded from the Si adatoms by 2.6 Å.

The bend of the molecule explains the invisibility of the carboxyl parts in PTCDA at constant-height scan, as shown in Fig. 2a. Only the more protruding central part of the molecule is imaged. Our Δ*f* image could be reproduced by a calculated map of electron density cut along a plane parallel to the Si(111)-(7 × 7) surface (see Fig. 2b). Electron density was high mainly at the five carbon rings at a constant-height. In previous theoretical calculations using the same molecule^24^, features in the electron density map were in good agreement with those in the Δ*f* map, as well as with the map of kinetic energy. The enhancement of kinetic energy as the tip approaches shows the origin of the Pauli repulsive force.

### Tip characterization by force measurements and calculation

**Figure 3:**
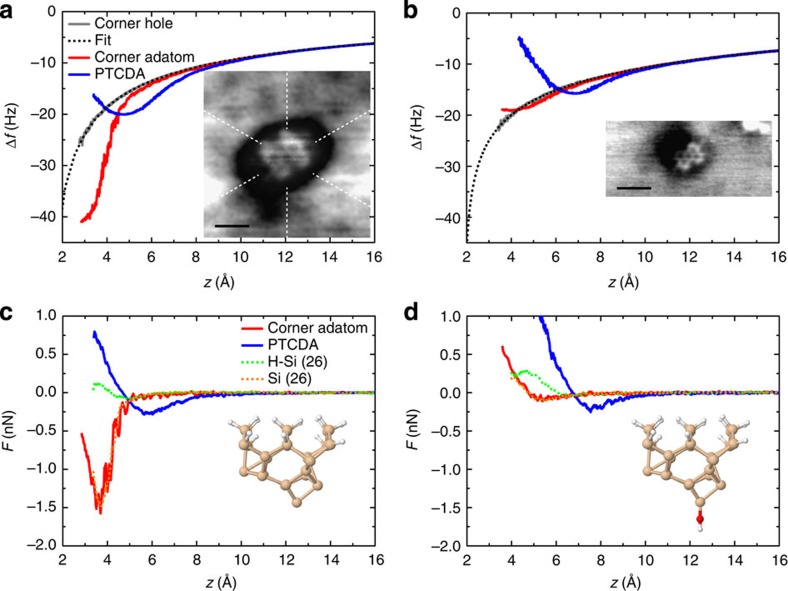
Tip identification by force measurements. (**a,b**) Δ*f*(*z*) curves measured over the centre of the PTCDA molecules (blue), corner adatoms (red) and corner holes (gray) using (**a**) a reactive tip and (**b**) an inert tip. Fitting curves for the long-range force contributions are shown by the dotted curves. The acquisition parameters were (**a**) *f*_0_=165,371.4 Hz, *k*=37.6 N m^−1^, *A*=133 Å, *V*_*s*_=*V*_c.p.d._=−280 mV and *Q*=23,000, (**b**) *f*_0_=156,719.7 Hz, *k*=29.6 N m^−1^, *A*=201 Å, *V*_*s*_=*V*_c.p.d._=−350 mV and *Q*=25,000. Corresponding AFM images are shown in the insets. Scale bar, 1 nm. (**c**,**d**) *F*(*z*) curves over the centre of the PTCDA molecules (blue) and corner adatoms (red), converted from the short-range part of the Δ*F*(*z*) curves from **a** and **b**. The *F*(*z*)curves over the H adsorbed on Si adatoms (green) and Si adatoms (orange) from our previous study^26^ are also shown. The tip models identified are shown in the insets.

We were able to obtain the chemical structure using Si cantilevers. Although CO tip functionalization is not currently possible at RT, we can characterize the tip state that would be capable of producing chemical structures in the molecule by force measurements. For this purpose, we carried out force spectroscopy using the same tip at three different sites, that is, the centre of the molecule, a corner Si adatom sufficiently far from the molecule, and a bare corner hole. The distance dependences of Δ*f* curves with two different types of tips are shown in Fig. 3a,b. Δ*f*(*z*) curves on the corner hole (gray curves), which corresponded to the long-range force, were fitted into an inverse power function. The fitting curves were subtracted from the Δ*f*(*z*) curves above the molecules (blue curves) and the Si adatoms (red curves). The obtained short-range part of the Δ*f*(*z*) curves was converted into the short-range forces (*F*(*z*)) using the conversion formula^25^ (see the solid blue and red curves in Fig. 3c,d). Similar to our previously reported study^26^, the tips in Fig. 3c,d could be classified as a reactive tip and non-reactive tip, respectively. They were characterized by the magnitude of the maximum attractive force on the Si adatoms. Reactive tips produce a strong chemical bonding force (>0.5 nN) on an Si adatom site, while non-reactive tips produce a weak physical force (<0.1 nN). This is partly because reactive tips have dangling bonds that hybridize with the dangling bonds on Si adatoms to form covalent bonds. Non-reactive tips do not have dangling bonds, that is, they are chemically inert. Clear atomic resolution of each Si adatom on the Si(111)-(7 × 7) surface can be obtained only with reactive tips. Nevertheless, we found that both types of tips could produce an image of the inner molecular structure by Pauli repulsion, as shown in the insets of Fig. 3a,b. This is also evident from the similarity of the *F*(*z*) curves on the molecule with the different types of tips (see the blue curves in Fig. 3c,d). In both types of tips, weak maximum attractive forces (approximately −0.27 nN) are obtained on the molecule before the tips enter the repulsive-force region (*F>*0).

This result was comparable to our previous findings on H-terminated Si adatoms on the Si(111)-(7 × 7) surface^26^. AFM images showed contrast corresponding to the repulsive force on H atoms with both types of tips^26^. For comparison, both types of *F*(*z*) curves previously measured on H-terminated Si adatoms are shown in Fig. 3c,d (green dotted curves). *F*(*z*) curves on Si adatoms measured by the same tips are also shown (orange dotted curves); these curves were duplicated from Fig. 6a,b in our previous paper^26^. The curves on Si adatoms in the present studies are in good correspondence with the previous ones, implying that the tip states are very similar (that is, the Si(001) dimer tip for Fig. 3c and OH-terminated dimer tip for Fig. 3d, as previously identified by DFT calculation). The formation of the OH-terminated tip originates from the residual H_2_O gas inside the ultrahigh vacuum chamber. The tip models are shown in the insets of Fig. 3c,d. The *F*(*z*) curves on the H sites measured by these reactive and non-reactive tips are similar. The maximum attractive forces on the H sites are tiny (<0.1 nN) for both types of tips. They are smaller than those on the PTCDA molecules, where the carbon atoms composing the centre rings contribute to the physical force.

The termination of the non-reactive tip by the –OH group can also explain the enhanced submolecular resolution of the AFM image shown on Fig. 3b, in comparison with the reactive counterpart shown in Fig. 3a. This effect can be attributed to the much smaller atomic radius of the frontier H atom in the case of –OH group in comparison with the apex Si atom on the reactive tip. The smaller apex atom can penetrate deeper into the interatomic voids (that is, benzene rings), providing higher submolecular contrast as was discussed in ref. 12.

We conclude that tip reactivity is not crucial for chemical structure imaging when the tip scans on the inert closed-shell molecule that is strongly bound on the semiconductor surface. We calculated *F*(*z*) on some points of the PTCDA molecule using the Si dimer tip model as described below. The maximal attractive force was small over all sites, including an edge oxygen atom bonded to an Si adatom, since the reactive orbital of the oxygen atom was terminated by the surface Si adatom. The molecule can be stably imaged even using a reactive tip. Our results are also consistent with a recent study of NTCDI molecules on the Si(111)-(7 × 7) surface at low temperature^11^,^27^.

Next, we used force spectroscopic data to further clarify the difference in tip height required for atomic resolution between the molecule and Si surface. In Fig. 2a, high resolution was obtained only on the molecule, while there was no atomic resolution on the Si(111)-(7 × 7) surface. In our *F*(*z*) curves acquired using a number of tips with both types of reactivity, the difference in the distances for maximum attractive force between molecules and Si adatoms was about 2 Å (see Fig. 3c,d). This can be explained by the topographic height difference between the molecule and Si adatom layer as seen in Fig. 2d. When the tip–sample distance for constant-height imaging was gradually decreased, the tip started to interact with the molecule first. At the repulsive region (*F*>0) on the molecule in Fig. 3c, the reactive tip reached the onset of the short-range force on Si adatoms. There, atomic resolution on Si adatoms could be obtained by attractive force contrast, as shown in the inset of Fig. 3a. On the other hand, the non-reactive tips could not provide clear atomic resolution on the Si adatoms by attractive force. It may have been expected that both types of tips would be capable of providing atomic resolution on Si adatom sites by repulsive interaction forces at very close distances, since such resolution was indeed recently obtained at low temperature^16^. However, we found that it was difficult to scan at such close distances at RT without tip changes.

## Discussion

**Figure 4:**
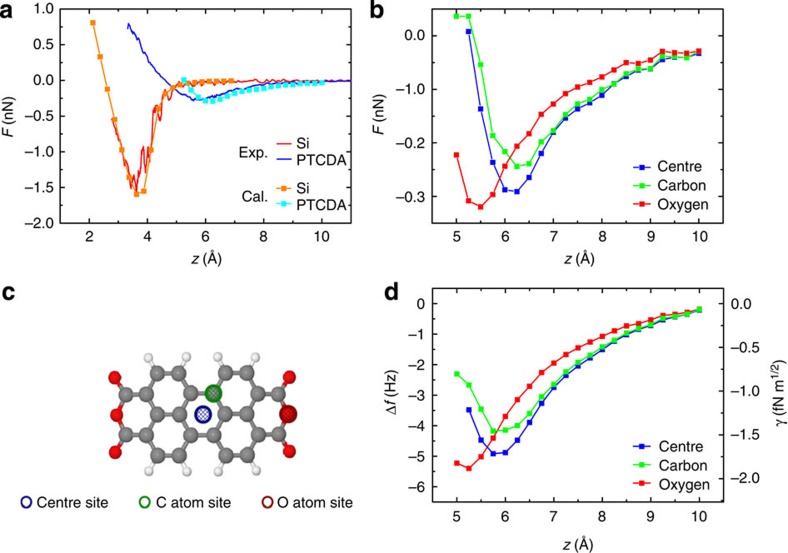
Theoretical force and frequency shift curves. (**a**) Theoretical *F*(*z*) curves over the centre of a PTCDA (light blue) and a corner adatom (orange). The model of an Si dimer tip shown in Fig. 3c is used. The corresponding experimental curves from Fig. 3c are shown: *F*(*z*) on PTCDA (blue) and Si adatom (red). The Van der Waals (vdW) force is taken into account for the calculations on the molecule. The theoretically derived maximum attractive force is around −0.29 nN, which matches the value obtained experimentally. Since the attractive force in the *F*(*z*) calculated without the vdW contribution is too small to reproduce the experimental results (not shown), the vdW interaction force dominates the attractive force on the molecule. (**b**) Theoretical *F*(*z*) curves over the centre of a PTCDA (blue), a carbon atom (green) and an oxygen atom (red). (**c**) A model of the PTCDA molecule showing the three sites used for the force calculations. (**d**) Δ*f*(*z*) curves converted from the *F*(*z*) curves shown in **b**. For the conversion, we used the same parameters as used for the experiments shown in Fig. 3a.

Theoretical short-range force *F*(*z*) curves on the centre of the molecule and Si adatoms are plotted in Fig. 4a. The corresponding experimental curves from Fig. 3c are also shown. As mentioned above, the Si dimer tip reproduced the experimental *F*(*z*) curve measured above the Si adatom (see the red and orange curves). In addition, the *F*(*z*) curve calculated on the molecule well reproduced the features of the experimental curve, such as the maximum attractive force (see the blue and light blue curves).

To gain more insight into the mechanism of the submolecular contrast, we analysed the differences among the short-range force *F*(*z*) curves calculated for three characteristic sites of the PTCDA molecule, that is, an oxygen (O), a hollow (h) and a carbon (C) site (see Fig. 4c). The calculated *F*(*z*) curves are shown in Fig. 4b. According to our DFT simulations, the van der Waals (vdW) interaction represented most of the attractive portion of the *F*(*z*) curves in far tip–sample distances. The vdW interaction provides a common attractive background with a larger maximum on the h-site than on the C-site. The characteristic bending of the molecule causes the largest attractive interaction over the O-site, which is shifted downward with respect to the central part of the molecule. At close distances, the Pauli repulsion between electronic clouds on the tip and sample prevails, and it is larger at the areas of higher electron density (for example, on top of the carbon atoms; see Fig. 2b). The presence of the Pauli repulsion further enhances the difference between the short-range forces *F*(*z*) calculated at the three characteristic sites. This effect is responsible for the observed submolecular contrast.

Next, we estimated the tip–sample distance, where the submolecular image was acquired. For this purpose, we compared the change of the normalized frequency shift *γ*^28^ among the three characteristic O, h and C sites between experiment and theory. From the high-resolution experimental image obtained with a reactive tip (inset of Fig. 3a), we could estimate that the *γ* values were −4.7, −4.5 and −5.25 [fN·m^1/2^] for the h, C and O-site, respectively. Thus we obtain following differences *γ*_*h-O*_=*γ*_*h*_–*γ*_*O*_=+0.55 [fN·m^1/2^] and *γ*_*h*–*C*_=−0.2 [fN·m^1/2^], respectively. These differential values were well reproduced in the theoretical curves *γ*_*h-O*_=+0.7 and *γ*_*h–C*_=−0.3 [fN·m^1/2^] at distance *z*=5.25 Å (see Fig. 4d). Therefore, we can conclude that the submolecular contrast over the central part of the molecule was achieved in the repulsive regime due to the difference in the attractive vdW and, Pauli repulsive interactions, with the latter making the major contribution.

Note that both the submolecular images acquired with the reactive and those acquired with the non-reactive Si-based tips show relatively blunt contrast, which matches the submolecular contrast observed on the PTCDA/Ag(111) surface in relatively far distances (see Fig. 2c in ref. 12). In the distance range, the distortion of a functionalized probe does not play any role and the contrast is completely driven by the difference in the force over different sites (hollow, atom). This concurs very well with the fact that we are able to achieve the submolecular contrast even with a relatively rigid, bare Si-tip. This leads to an important question, namely, can a sharp submolecular resolution be realized using Si-based tips with an intentionally functionalized group at RT? In principle, such resolution could be hampered by two factors: (i) the presence of inherent noise due to thermal fluctuation, and/or; (ii) the instability of a functional group on the tip apex. Thus the further improvement of the submolecular resolution is open to question. We believe that this question will be addressed in the near future.

In conclusion, we successfully obtained the chemical structure of single PTCDA molecules (1) on a semiconducting Si surface; (2) at room temperature; (3) using soft Si cantilevers; and (4) under oscillation at large amplitude. Force spectroscopic measurements showed that high resolution could be obtained independently of the tip reactivity. The DFT calculations clarified the geometry of the molecule and the origin of the high-resolution AFM image reflecting the electron density map over the molecule. Our less-restrictive method for submolecular imaging opens a new route for further applications using AFM. We believe that this work will stimulate an effort to achieve chemical structure imaging of single molecules even in ambient and liquid environments using high-resolution AFM based on Si cantilevers^29^,^30^.

## Methods

### Procedures for high-resolution molecular imaging by AFM

We obtained high-resolution molecular AFM images according to the following protocol. (1) Search for PTCDA molecules that are adsorbed at the corner-hole sites by STM under a dynamic mode. (2) Switch from the operation mode to the AFM topographic mode. Set the tip–surface distance to be large so that AFM will not have atomic/molecular resolution. (3) Start the constant-height scan in the retrace mode, in which each fast-scan line is scanned twice^31^. Close the distance feedback loop in the first scan and open it in the second scan. In the first scan, the tip–surface distance will be large and thus the obtained atomic-scale contrast will not have surface features. Perform the second scan along a line parallel to the linear fitting of the first scan while recording Δ*f*. The tip–surface distance can be controlled by offset from the first scan height. (4) Gradually increase the offset to reduce the tip–surface distance for the second constant-height scan until submolecular resolution is obtained on the molecules. The tip states will change or the contrast will not be clear or the molecule will be moved at very small tip–surface distances at RT. Then, modify the tip state using the following procedures. (i) Scan the surface by dynamic STM. To modify the tip, gently poke it into the surface by controlled force distance spectroscopy, reaching the strong repulsive regime^32^. Bring the tip to the clean Si region and open the distance feedback. Then, allow the tip to approach the surface by a certain displacement while recording the tunnelling current and Δ*f*. If no abrupt jump is observed in either channel, then repeat the procedure with increasing displacement until an abrupt change of signals is observed in accompaniment with the tip change. (ii) Next, rescan the surface by dynamic STM to check the tip quality. If the tip is unstable or shows multi-tip features, then high-resolution molecular imaging cannot be achieved by AFM. Then, we again modify the tip by returning to step (i). (iii) When the tip is optimized in STM, restart the molecular imaging procedure at step (1) above. AFM images, which were obtained before Fig. 2a and Fig. 3a were acquired, are shown in Supplementary Fig. 1.

### Consideration of the signal-to-noise ratio for AFM imaging

Chemical structures can be successfully obtained at RT using Si cantilevers oscillated even at large *A*. To achieve such high resolution, tiny force differences reflecting electron density should be detected through the AFM observable, which is Δ*f*. A signal-to-noise ratio (S/N) in Δ*f* for constant-height AFM imaging has been previously discussed^21^. Here we show that a soft Si cantilever with an optical interferometer has a higher S/N than the qPlus sensor popularly used. The Δ*f* signal is proportional to *f*_0_/*k*, where *f*_0_ and *k* are the resonance frequency and stiffness of a cantilever, respectively. This factor in our Si cantilever (*f*_0_=160 kHz, *k*=30 N/m) is 400 times larger than that in a typical quartz cantilever (*f*_0_=23 kHz, *k*=1,800 N/m). In addition, the Δ*f* signal increases with decreasing *A*. Δ*f* is proportional to 1/*A*^1.5^ at large *A* approximation^33^. Since a soft Si cantilever should be oscillated at large *A* to avoid instability, an Si cantilever has a smaller signal than a quartz cantilever. This factor for an Si cantilever oscillated at *A*=20 nm becomes at most 2,800 times smaller than that of a quartz cantilever oscillated at *A*=0.1 nm. On the other hand, the deflection noise in Δ*f* becomes proportional to *n/A*, where *n* is the noise density of the defection sensor^2^. Our optical interferometer has a much smaller *n* (15 fm/
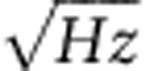
)^22^ than the typical qPlus sensor (60 fm/
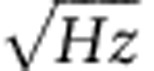
) at RT^34^. The noise in the Si cantilever with an optical interferometer is 800 times smaller than that in a qPlus sensor. In short, the Si cantilever with an optical interferometer has a better S/N than the qPlus sensor by two orders of magnitude. A large S/N in Δ*f* is essential for constant-height AFM imaging at RT because the scan speed must be fast enough under non-negligible thermal drift.

### Calculation method

The simulated *F*(*z*) curves with the clean Si dimer tip and the OH-terminated dimer tip were calculated using VASP (Vienna Ab initio Simulation Package)^35^. VASP is a density functional theoretical code that implements a pseudopotential plane wave method. We used Vanderbilt ultrasoft pseudopotentials^36^. The softer version of the oxygen pseudopotentials supplied with VASP was used. The exchange-correlation function was described within the PW91-generalized gradient approximation^37^. We used the semiempirical approach by Grimme^38^ for the vdW calculations. The Si(111) surface was represented by a slab consisting of the adatom layer and two more 7 × 7 Si(111) bi-layers below. The bottom Si atomic layer was passivated with hydrogen atoms. When geometry optimization was performed, the atoms of the PTCDA molecule and 24 Si atoms close to the O–Si bonds were allowed to relax, while all other Si atoms and the saturating hydrogens were kept at fixed positions. We used well-tested Si-based tips^39^, where 15 Si atoms at the base of the tip and saturating hydrogen atoms were kept fixed during relaxation. The geometry optimization was converged down to a precision of 10^−5^ meV in total energy. The Brillouin zone sampling was restricted to the central (Γ) point. The basis set cutoff for wave function expansion was set to 286.7 eV of kinetic energy, which is the recommended value for carbon pseudopotential (the hardest pseudopotential of the elements used). The maximal wave vector for charge density and local (Hartree) potential expansion was set to twice the maximal wave vector for the wave function expansion (to avoid aliasing effects).

## Additional information

**How to cite this article:** Iwata, K. *et al*. Chemical structure imaging of a single molecule by atomic force microscopy at room temperature. *Nat. Commun.* 6:7766 doi: 10.1038/ncomms8776 (2015).

## Supplementary Material

Supplementary Information Supplementary Figure 1
