# Supplementary material for: Chemical structure imaging of a single molecule by atomic force microscopy at room temperature
Source: Nat Commun. 2015 Jul 16;6:7766. doi: 10.1038/ncomms8766 (PMC4518281; doi:10.1038/ncomms8766)
Supplement: Supplementary Information — Supplementary Figure 1 [file ncomms8766-s1.pdf]

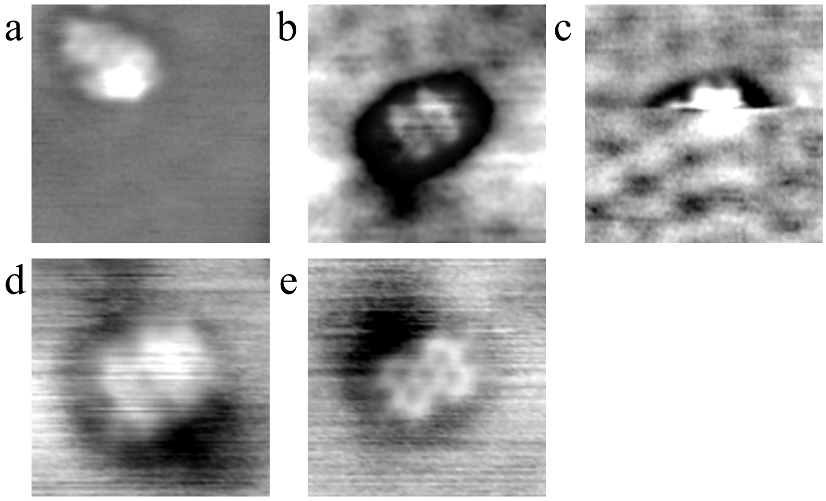

Supplementary Figure 1

Tip optimization processes for high resolution AFM imaging

a-c Constant height AFM images of a single PTCDA molecule adsorbed on the Si(111)-(7×7) surface using a reactive tip. Since image (a) showed multi tip feature, the tip was modified by the controlled tip-sample contact, then we obtained clearer AFM contrast shown in (b) that is the same as Fig. 3(a) in the main text. After that, the tip scan from top to bottom at closer tip-surface distance caused manipulation of the molecule as shown in (c). **d-e** Constant height AFM images of a single PTCDA molecule adsorbed on the Si(111)-(7×7) surface using a non-reactive tip. Since image (d) had unclear contrast, the tip was modified by the controlled tip-sample contact, then we obtained clearer AFM contrast shown in (e) that is the same as Fig. 2(a) in the main text.
